# Atypical Presentation of Pheochromocytoma With Persistent Fever of Unknown Origin: A Case Report

**DOI:** 10.7759/cureus.102168

**Published:** 2026-01-23

**Authors:** Anas E Ahmed, Ghada A Alqarni, Abdulrahman M Aloufi, Reem F Hawkash, Yasmeen M Alhaji

**Affiliations:** 1 Community Medicine, Jazan University, Jazan, SAU; 2 College of Medicine, University of Bisha, Bisha, SAU; 3 College of Medicine, Hail University, Hail, SAU; 4 College of Medicine, Najran University, Najran, SAU; 5 College of Medicine, Universiti Sains Malaysia, Kelantan, MYS

**Keywords:** adrenal incidentaloma, adrenal tumor, atypical presentation, fever of unknown origin, laparoscopic adrenalectomy, metanephrines, mibg scintigraphy, neuroendocrine tumor, pheochromocytoma, systemic inflammatory response

## Abstract

Pheochromocytoma is a rare neuroendocrine tumor classically associated with episodic adrenergic symptoms and hypertension, but its clinical presentation is highly variable and may be misleading. Fever is an uncommon manifestation and is rarely the dominant presenting feature, which can result in diagnostic delay and extensive evaluation for infectious, inflammatory, or malignant causes. We report the case of a middle-aged woman who presented with prolonged fever, weight loss, and systemic inflammatory features without hypertension or classic catecholamine-related symptoms. Extensive investigations for infectious, autoimmune, and malignant etiologies were unrevealing. Cross-sectional imaging incidentally identified a right adrenal mass, which on further evaluation demonstrated imaging and biochemical features consistent with pheochromocytoma. Functional imaging supported the diagnosis and excluded disseminated disease. The patient underwent appropriate preoperative medical preparation followed by laparoscopic adrenalectomy, with rapid postoperative resolution of fever and inflammatory markers and sustained clinical recovery on follow-up. This case highlights an unusual inflammatory presentation of pheochromocytoma and emphasizes the importance of maintaining a broad differential diagnosis in patients with fever of unknown origin. Endocrine tumors should be considered in the evaluation of persistent unexplained fever, particularly when an adrenal lesion is identified, even in the absence of classic hormonal symptoms. Early recognition is essential, as timely surgical intervention is curative and prevents potentially life-threatening complications associated with undiagnosed pheochromocytoma.

## Introduction

Pheochromocytomas are rare neuroendocrine tumors arising from chromaffin cells of the adrenal medulla and are characterized by autonomous catecholamine secretion [1]. The classic clinical triad includes episodic headache, diaphoresis, and palpitations, often accompanied by paroxysmal or sustained hypertension [2]. However, the clinical spectrum of pheochromocytoma is highly variable, and atypical presentations may occur, leading to diagnostic delay [1,2]. Fever is an uncommon manifestation and is rarely the dominant presenting symptom [1-3]. When present, it is often attributed to tumor necrosis, catecholamine-induced hypermetabolism, or cytokine release, and may mimic infectious, inflammatory, or malignant conditions [3,4].

Fever of unknown origin (FUO) remains a diagnostic challenge and encompasses a wide range of infectious, autoimmune, neoplastic, and miscellaneous causes [3]. Endocrine etiologies are rarely considered in the initial evaluation of FUO, particularly in the absence of classic hormonal symptoms [4]. Consequently, pheochromocytoma presenting primarily as FUO is frequently overlooked, resulting in prolonged morbidity and unnecessary investigations [2-4]. This case highlights an unusual presentation of a right adrenal pheochromocytoma manifesting as persistent fever with systemic inflammatory features and no adrenergic symptoms, underscoring the importance of maintaining a broad differential diagnosis and considering endocrine tumors in patients with prolonged unexplained fever.

## Case presentation

A 42-year-old woman with no significant past medical history was referred to our tertiary care center for evaluation of a three-month history of persistent fever of unknown origin. The fever was intermittent, predominantly evening in onset, reaching up to 38.5-39.0°C, and was associated with night sweats, malaise, anorexia, and unintentional weight loss of approximately 6 kg. There was no associated cough, hemoptysis, urinary symptoms, abdominal pain, diarrhea, rash, joint pain, or recent travel. She denied palpitations, episodic headaches, diaphoresis, or anxiety attacks. There was no history of hypertension, diabetes, tuberculosis exposure, or malignancy. She was not taking any regular medications and had no family history of endocrine tumors or hereditary syndromes.

On physical examination at admission, the patient appeared fatigued but was alert and oriented. Her temperature was 38.2°C, blood pressure was 128/76 mmHg, heart rate was 96 beats per minute, respiratory rate was 18 breaths per minute, and oxygen saturation was 98% on room air. There was no pallor, jaundice, lymphadenopathy, or peripheral edema. Cardiovascular and respiratory examinations were unremarkable. The abdomen was soft and non-tender with no palpable masses or organomegaly. There were no skin lesions, flushing, or signs suggestive of connective tissue disease. Neurological examination was normal.

Initial laboratory investigations demonstrated normocytic normochromic anemia (hemoglobin 10.4 g/dL), leukocytosis (12.8 × 10⁹/L) with neutrophil predominance, elevated erythrocyte sedimentation rate (ESR, 78 mm/hour), and elevated C-reactive protein (CRP, 112 mg/L). Liver and renal function tests were within normal limits. Blood cultures drawn on three separate occasions were negative. Serologic tests for human immunodeficiency virus (HIV), hepatitis B and C viruses, Brucella species, Epstein-Barr virus, cytomegalovirus, and autoimmune markers, including antinuclear antibody and rheumatoid factor, were negative. Urinalysis was normal, and urine cultures were sterile (Table 1). A chest radiograph was unremarkable.

**Table 1:** Summary of laboratory investigations at presentation and during diagnostic workup This table summarizes the hematological, biochemical, immunological, and urinary laboratory findings obtained during the patient’s initial evaluation. Reference ranges correspond to standard adult values.

Parameter	Result	Unit	Reference range
Hemoglobin	10.4	g/dL	12.0–16.0
Hematocrit	31.8	%	36–46
Red blood cell count	3.7	×10¹²/L	4.0–5.2
Mean corpuscular volume	86	fL	80–96
Mean corpuscular hemoglobin	28	pg	27–33
Mean corpuscular hemoglobin concentration	32	g/dL	32–36
Red cell distribution width	14.8	%	11.5–14.5
White blood cell count	12.8	×10⁹/L	4.0–10.0
Neutrophils	78	%	40–75
Lymphocytes	14	%	20–45
Monocytes	6	%	2–10
Eosinophils	2	%	1–6
Basophils	0.5	%	<1
Platelet count	420	×10⁹/L	150–400
C-reactive protein	112	mg/L	<5
Erythrocyte sedimentation rate	78	mm/hr	<20
Ferritin	480	ng/mL	15–150
Procalcitonin	0.08	ng/mL	<0.5
Urea	28	mg/dL	15–40
Creatinine	0.8	mg/dL	0.6–1.2
Alanine aminotransferase	22	U/L	7–40
Aspartate aminotransferase	24	U/L	10–40
Alkaline phosphatase	88	U/L	40–130
Total bilirubin	0.6	mg/dL	0.2–1.2
Sodium	138	mmol/L	135–145
Potassium	4.1	mmol/L	3.5–5.0
Chloride	102	mmol/L	98–106
Bicarbonate	24	mmol/L	22–28
Calcium	9.2	mg/dL	8.5–10.5
Plasma free metanephrines	Elevated	—	Normal
Plasma free normetanephrines	Elevated	—	Normal
24-hour urinary catecholamines	Elevated	—	Normal
Serum cortisol	Normal	µg/dL	5–25
Plasma aldosterone	Normal	ng/dL	4–31
Plasma renin activity	Normal	ng/mL/hr	0.5–4.0
Thyroid stimulating hormone	2.1	mIU/L	0.4–4.5

**Figure 1:**
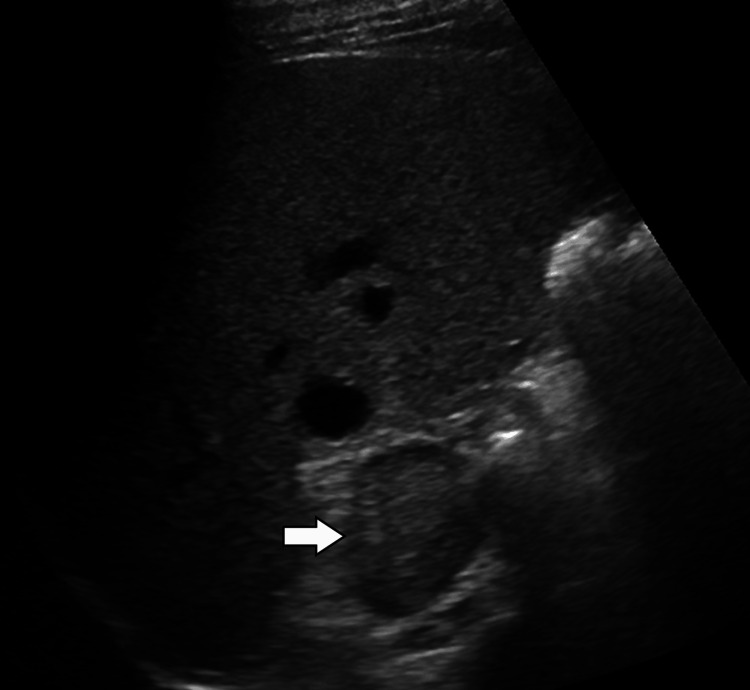
Ultrasound of the right adrenal gland Greyscale ultrasound image of the right adrenal region demonstrates a well-defined, hypoechoic soft tissue mass (arrow). No obvious cystic or calcific components are seen.

**Figure 2:**
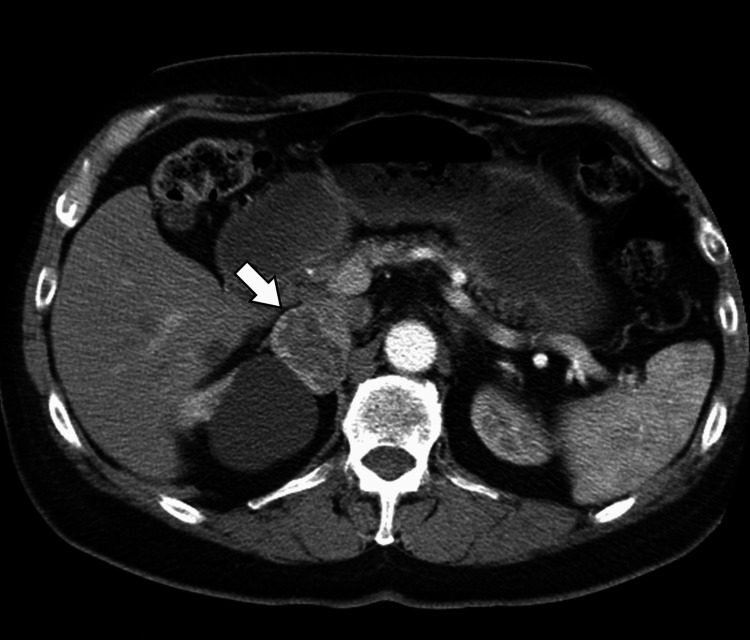
Contrast-enhanced axial CT of the abdomen Axial contrast-enhanced computed tomography (CT) image shows a large, heterogeneous soft tissue lesion in the right adrenal gland (arrow) with areas of low attenuation and mild peripheral enhancement.

Given the persistent inflammatory markers and fever without an identifiable source, further imaging was pursued. Abdominal ultrasonography revealed a well-defined, heterogeneously hypoechoic mass measuring approximately 5.5 × 4.8 cm in the right suprarenal region, separate from the liver and kidney (Figure 1). Contrast-enhanced computed tomography (CT) of the abdomen showed a 6.0 × 5.2 cm right adrenal mass with heterogeneous enhancement, central areas of low attenuation suggestive of necrosis, and no evidence of local invasion or lymphadenopathy (Figure 2).

**Figure 3:**
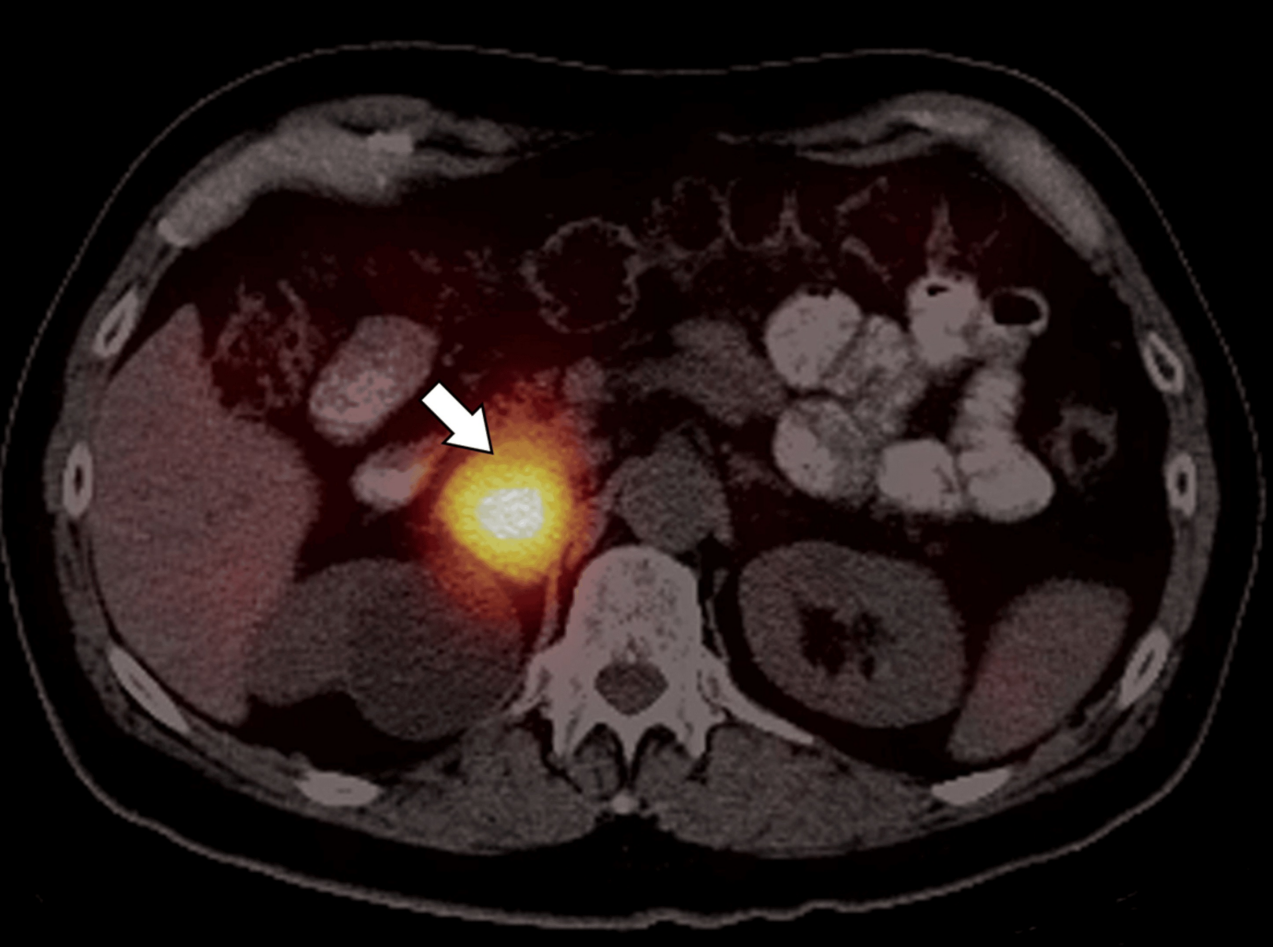
Axial ¹²³I-MIBG scintigraphy showing adrenal lesion uptake Axial iodine-123 metaiodobenzylguanidine (¹²³I-MIBG) single-photon emission computed tomography (SPECT) image demonstrates intense radiotracer uptake corresponding to the right adrenal lesion (arrow), consistent with a pheochromocytoma.

Biochemical evaluation for adrenal hormone excess revealed elevated plasma free metanephrines and normetanephrines, with 24-hour urinary fractionated catecholamines also significantly elevated. Serum cortisol, aldosterone, and renin levels were within normal limits. An iodine-123 metaiodobenzylguanidine (¹²³I-MIBG) scintigraphy scan showed increased radiotracer uptake localized to the right adrenal mass with no evidence of metastatic disease (Figure 3).

The patient was started on preoperative alpha-adrenergic blockade with phenoxybenzamine, gradually titrated over two weeks to achieve adequate blood pressure control and prevent intraoperative hypertensive crises, followed by cautious beta-adrenergic blockade. Despite the absence of classic paroxysmal symptoms, she tolerated the preparation well. Subsequently, she underwent elective laparoscopic right adrenalectomy without intraoperative complications. Intraoperative blood pressure fluctuations were minimal and controlled pharmacologically.

Postoperatively, the patient had an uneventful recovery. Her fever resolved completely within 48 hours of surgery, and inflammatory markers gradually normalized over the following two weeks. She was discharged on postoperative day five in stable condition without antihypertensive medications. At three-month and six-month follow-up visits, the patient remained asymptomatic, afebrile, and had regained weight. Repeat plasma metanephrines were within normal limits.

## Discussion

Pheochromocytomas are rare catecholamine-secreting neuroendocrine tumors with an estimated annual incidence of two to eight cases per million and are most commonly diagnosed in the fourth to fifth decades of life [2,5]. While the classical clinical phenotype consists of episodic headache, palpitations, diaphoresis, and paroxysmal hypertension, the clinical expression is remarkably heterogeneous [1-8]. Atypical or non-specific manifestations may predominate, including weight loss, anxiety, gastrointestinal symptoms, or glucose intolerance, and in rare instances, systemic inflammatory features such as fever may be the presenting complaint [3,5]. This phenotypic variability contributes substantially to delayed diagnosis and misattribution to infectious, inflammatory, or malignant processes.

Fever in pheochromocytoma is uncommon and poorly understood, but several mechanisms have been proposed [7,8]. Tumor necrosis and hemorrhage may provoke inflammatory responses with the release of pyrogenic cytokines such as interleukin-6 (IL-6), which is elevated in some pheochromocytomas, and correlates with fever, elevated CRP, and anemia of chronic disease [2,7]. Catecholamine excess itself can also increase metabolic rate and thermogenesis, potentially contributing to hyperthermia [3,5]. In some cases, pheochromocytomas have been described as functioning inflammatory tumors with cytokine secretion independent of infection [2,7]. This inflammatory phenotype can dominate the clinical picture and obscure the endocrine nature of the disease, as occurred in the present case.

The diagnostic challenge in this case lies in the absence of classic adrenergic symptoms and normotension, which are commonly perceived as prerequisites for considering pheochromocytoma [1,2]. However, up to 10-15% of pheochromocytomas are normotensive, and paroxysmal symptoms may be subtle or absent [3,5]. This highlights the importance of maintaining a broad differential diagnosis in patients with FUO and systemic inflammation, particularly when imaging reveals an adrenal incidentaloma [6,7]. In such scenarios, biochemical testing for catecholamine excess is essential before any invasive procedures.

Radiologically, pheochromocytomas typically appear as well-circumscribed adrenal masses with heterogeneous enhancement, high signal intensity on T2-weighted MRI sequences, and avid uptake on MIBG scintigraphy [4-8]. In this case, the concordance of CT, MRI, and functional imaging strongly supported the diagnosis and excluded metastatic disease. Functional imaging also played a critical role in confirming tumor activity and localization.

Surgical resection remains the definitive treatment for localized pheochromocytoma, with preoperative alpha-adrenergic blockade being essential to reduce perioperative cardiovascular complications [2,3]. Even in normotensive patients, preoperative preparation is recommended because catecholamine release during tumor manipulation can precipitate hypertensive crises, arrhythmias, or cardiomyopathy [3-5]. The rapid postoperative resolution of fever and normalization of inflammatory markers in this patient further support the tumor as the source of systemic inflammation [2-4].

## Conclusions

This case underscores that pheochromocytoma, although classically associated with paroxysmal adrenergic symptoms and hypertension, can present atypically as fever of unknown origin with systemic inflammatory features and without overt catecholamine-related manifestations. Such presentations may lead to significant diagnostic delay and extensive unnecessary investigations if endocrine causes are not considered. Clinicians should maintain a high index of suspicion for pheochromocytoma in patients with persistent unexplained fever, elevated inflammatory markers, and an adrenal mass, even in the absence of hypertension or classic symptoms. Early recognition and appropriate biochemical and imaging evaluation are crucial, as timely surgical management is curative and can result in rapid resolution of systemic symptoms.
